# Blackness unbound: autoethnography, culture, and global blackness through the Triple Helix Model

**DOI:** 10.3389/fspor.2026.1814553

**Published:** 2026-05-14

**Authors:** A. Lamont Williams

**Affiliations:** Sport Administration, University of Cincinnati, Cincinnati, OH, United States

**Keywords:** autoethnography, blackness, Italy, James Baldwin, Kendrick Lamar, Triple Helix Model (THM)

## Abstract

In this cultural autoethnography, the author examines how global travel (re)shapes Black identity, embodiment, and racial consciousness through a sport-influenced lens. Drawing on personal experiences traveling in Italy as a Black sport professor, the author blends personal narratives, excerpts from James Baldwin, music lyrics from Kendrick Lamar, and the theoretical lens of the Triple Helix Model to explore how universities, industries, and governmental systems collectively shape internalized understandings of Blackness. The manuscript interrogates how racialized expectations formed within American sport and society travel abroad as part of an internalized racism of mind, influencing perceptions of safety, belonging, and self-representation for Black people. Encounters in Italian public spaces—particularly those mediated through global sporting cultures such as football—illuminate the tensions between stereotype, fear, and cultural curiosity. The work demonstrates how international travel can reveal and disrupt internalized racism, offering pathways toward decolonizing the mind and reimagining Blackness beyond U.S. sociopolitical confines. Ultimately, this study contributes to global sport scholarship by foregrounding how Blackness is lived, negotiated, and transformed across cultural borders

This past, the Negro's past, of rope, fire, torture, castration, infanticide, rape; death and humiliation; fear by day and night. Fear as deep as the marrow of the bone; doubt that he was worthy of life, since everyone around him denied it; sorrow for his women, for his kinfolk, for his children, who needed his protection, and whom he could not protect; rage, hatred, and murder, hatred for white men so deep that it often turned against him and his own, and made all love, all trust, all joy impossible–this past, this endless struggle to achieve and reveal and confirm a human identity, human authority, yet contains, for all its horror, something very beautiful. I do not mean to be sentimental about suffering–enough is certainly as good as a feast–but people who cannot suffer can never grow up, can never discover who they are. (Baldwin, 1964, p. 66) (1).

***

## Proem: on cultural autoethnography and blackness

The cultural autoethnography presented here documents a stream of critiques, conflictions, and cultural connections being made through reflections regarding identity and racial consciousness in varying spaces. As a Black professor, traveler, and former athlete born and raised in/by America, I began this exposition with a quote from James Baldwin to show the ways that Black history (in America) can exemplify the idea of beauty in the struggle. In an identical way, my personal past is ripe with many conflicting understandings and contradictory experiences–and it wasn't until I traveled to the country of Italy that my feelings, thoughts, academic understandings, and cultural artifacts began to synchronize. Although I have had thoughts of this nature before (and subsequently tried to connect them in the past), it would not be until traveling to a country for pure explorational (or perhaps, vacational) reasons that I would begin to see how my experiences are informed by a collection of facets in my life–connected by my Black skin.

During my trip, I built on previous autoethnographic works (2) while “abroad” (that is, away from my primary country of residence; America) and once again, utilized the notes application of my iPhone to take notes during periods of downtime, exercise, and social engagements (or lack thereof). Whether consciously or subconsciously, I took note of everything that I saw, how people looked at me, how I perceived spaces and their reception of me, how my Blackness shifted certain spaces, and the paradox of Blackness outside of America. I admittedly tend to think of things through a racialized lens (in part, due to the racial hangover that is American culture), and as I continued my travels, I noticed that my best moments of clarity and conceptual connection to experiences came as the result of listening to music before, during, or after events. As a fan of Compton's Kendrick Lamar (an American artist), I listened to Kendrick's music and, throughout my stay in Italy, experienced various spiritual moments of connection and cultural clarity regarding the multifaceted nature of my Black skin (disclaimer: this article includes lyrics from Kendrick Lamar's songs, and all rights and ownership belong to Kendrick Lamar).

From a very young age, I have always been largely influenced by two things: sports and music. Not only is this a well-known stereotype for young, Black boys who come up in “urban” places (3) like that of where I grew up (St. Louis, Missouri), but for many years it was considered the two most prominent options for an amicable career path that hopefully keeps us out of jail (or alive in general). Those two career paths, which include being a musician (more specifically, a singer or rapper) or an athlete, exist in many cities across the United States and have become central to both expectations and identity development for young, Black boys. My personal history is much more complex, especially by way of family history, interests, and involvement; each layering expectations (and contradictions) that I wouldn't fully understand until much later.

I was surrounded by sports and music when I was a child, and oddly enough, not much has changed. Every Black man I saw was into music and sports. My grandfather used to run track, play baseball, and would later become a traveling musician with his singing group, *The Golden Truelights.* My father was a former basketball player (I only know this from photos and storytelling) and eventually would partake in various singing activities later in life. My uncle was a former NFL running back who then turned to music as well. That is not to say that *all* the Black men from the “hood were athletes turned singers”, but to say that the most glaring examples of people of which I took a following from were both athletically and musically inclined. Even when thinking about my own experiences, it was still always centric to sport and music. In high school, I would join my friends in creating musical songs (which we called raps) or participating in freestyle circles where people like my friend Demarcus would “ask for a beat” and freestyle over instrumentals until someone felt the urge to jump in. This took place anywhere and everywhere that we could gather, from living rooms and backyards to rooftops and street corners. When we weren't rapping together or listening to music while huddled around our boomboxes, we were playing basketball or participating in some sort of sporting activity–with music playing in the background. It was also not uncommon to see guys “running routes” with their Walkman's attached to their hips, nor was it rare to see others slam dunking and watching their cassette players crash to the ground from the sheer biomechanical force of the human body exploding to the rim and “posterizing” someone. That was the norm for Black boys when/where I grew up: music and sports. When I would go home, I would watch as my grandparents slow-danced and two-stepped to Al Green's greatest hits and wait for my mom to ask me to join her in the festivities (since I've always loved to dance). Once the dance was over, we would all pile onto the family couch and watch whatever sporting event that was on TV. Sport and music were all that I had ever known, and both were undeniably instrumental in my identity development.

Given my family history and the cultural emphasis on sport and music, it became surprising that very early in my doctoral studies, I deeply questioned the general value, love, and appreciation that people (and myself) have for sport [as a (a)vocation, academic discipline, or means of entertainment]. During that time, I existed in a space where most of the scholars rarely attended university (or professional) sporting events, rarely spoke of the “positives” of sport (outside of financial prowess), or even exuded a love for sport that I had once known as an athlete, scholar, and consumer. I was reminded of this trepidation when I traveled to the country of Italy and reunited with a friend (who does not work in sport). Immediately, the feelings of confusion–related to my thoughts of Blackness–returned and I started to think intimately; “what am I missing?”

Why sport?

Why study sport?

 and be so critical, as if sport is supposed to change the world

  …*my* world

 well, it has changed my world, sort of.

  but how can it change “the” world?

   …this world?

…when many people in this (scholarly) world also don't seem to enjoy *sport* itself.

Why then…am I still attached to this…thing?

 As a former Black athlete, is this the Black struggle that must be?

Maybe it is the Black athlete in me that drives the attachment to the world of sport…

Or perhaps it's the age-old Black tales of masculinity that keep me chained to a world that never truly accepts me

 unless I'm competing

  …and winning?

Much like the days of being a Black athlete

  …am I simply living a dream deferred?

    One that will always consume and rarely fulfill?

  One that will never allow me to just exist without performing?

    Is there such a thing?

      ..such a world?

        …for a Black man?

***

It was in that moment that I realized two things: (1) to say sport mirrors society does not tell the whole story, and (2) my double-consciousness as a Black man (who is a sport professor) has created a sort of never-ending social anxiety that may be better explained through a collective assessment of the spaces which reinforce my understanding (and confusion) of Blackness; universities, governments, and industries.

In this way, I use cultural autoethnography, inclusive of lyrics from Compton rapper Kendrick Lamar as cultural artifacts, to explore the multifaceted nature of Blackness for Black academics (in this case, applied to sport academics). I utilize the Triple Helix Model (THM) as a theoretical framework to explain the ways that universities, companies, and governments have primarily been focused on revenue generation and innovation–while ignoring how it feels for Black people to live within those systems. I am utilizing my own stories, experiences, and lived realities to show how these structures not only impact Black identity and belonging, but also how they can lead to social isolation, fragmented understanding of global cultures, and overall exclusion from spaces that don't appear to be constructed for the inclusion of Black people. I will lean on the teachings of James Baldwin to aid in the construction of understandings related to Blackness, and through an application of THM, expose how the continued ideas of race-neutrality by all parties has stifled identity development, created a landscape of internalized racism for Black people, and ultimately allowed for the subconscious adoption of colonized mentalities for Black people. Conversely, it is through the traveling of the world and connection to global cultures that Black people in America can release themselves from the shackles of internalized racism, decolonize our minds, and begin to live as truly free beings who are not always defined by the color of our skin. This approach not only offers a theoretical contribution by showing how Triple Helix spaces are also racialized (and therefore shapes Black identity development for those involved), but also makes a methodological contribution by way of using a cultural autoethnography with Black cultural texts (i.e., lyrics from Kendrick Lamar, writings from James Baldwin, etc.) that opens the door for innovation from other authors who may also be exploring Blackness on a global scale.

***

## Triple-Helix model–and dilemma

Growing up, living, and learning in America is a tough conundrum to navigate, especially when many young Black boys are taught from the moment we can remember to be on guard at all times. No matter how much our parents prepare us, most of us understand that even they cannot prepare us for what will happen when we are inadvertently abused, ridiculed, called a n*****, demoralized, harassed by police, or even killed because of our Black skin.

As early as I can remember, I was taught not to whistle at anyone because of what happened to Emmett Till…or trust police because of what they did to Rodney King

My momma taught me to “get your education and stick to your books”, because knowledge is the one thing they can never take from a Black man.

My momma also taught me to be a winner; we play sports–and we win.

I was taught that as a Black boy, my knowledge should also include the law and knowing my rights.

…but never was I told…that I had the freedom to choose my life and define Blackness for myself. This could be for several reasons, but no matter where I went, it was made quite clear that my Black skin would be a defining feature regarding how my presence in those spaces would ultimately pan out. Of course, my mother made it clear that I could “be whatever I set my mind to,” but even then, I was aware that the outside world didn't function that way. I would learn (very early in life) how the world treated Black boys, but I did not learn the true extent of Blackness, Black Masculinity, and the concept of Black Misandry until my mid-20's when I started doctoral studies. It was during that time that I also realized that the more I learned about Blackness and Black studies–the more trapped I felt. I was fortunate to be introduced to Critical Race Theory at the beginning of my studies, but with that new knowledge comes the weight of understanding that many laws and policies are not written with the intent of Black inclusion regarding life, liberty, and the pursuit of happiness. I was also introduced to scholarship related to Black Masculinity, which by extension opened the door for the study of Black Misandry. These concepts were great to know, but with it came the weight of understanding that no matter where I went–I would always be Black and presumed subpar.

**Figure 1:**
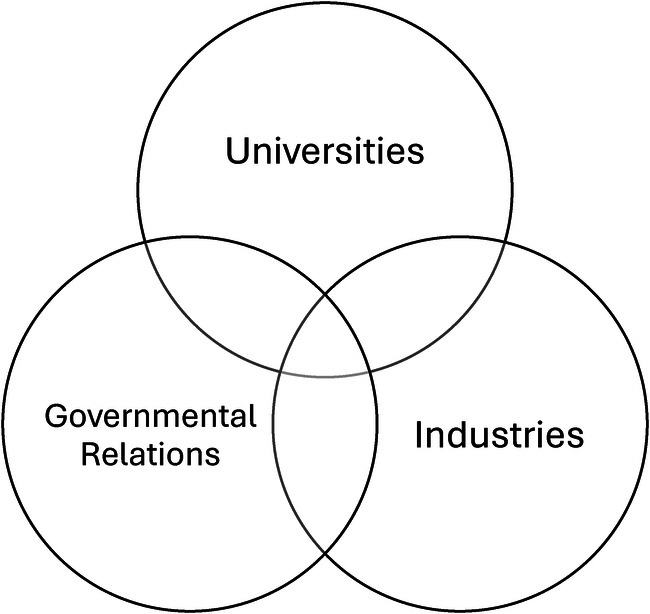
Balanced THM (4).

Possessing an intimate understanding of those concepts seemed to close in on me, as I was then presented with the weight of understanding that my Black body as a physical specimen meant I would be treated differently–or mistreated altogether for the sake of my existence. Though it would be a tough task as a boy coming from a working-class household, I made it my personal mission to “get out” and go to university. Somewhere. Somehow. It didn't matter what the cost, I would make it work–just as many before me had done. I was thankful that my critical studies in sport informed me on the “waves of athlete activism” and the ways that Black athletes have been mistreated just as much as our Black counterparts in general society–except the mistreatment was a direct result of us being “good” at sports and “beating white people”. Put differently, I learned that the better I (and those who look like me) are at sport (or, academics), the more that many people would hate us for it because we would be inadvertently winning over white people (which society deemed unacceptable). With these thoughts in mind, my travels to Italy brought about a new level of awareness, as I began to interrogate the major spaces/places that have influenced my life this far–and strangely, they fell within three subgroups that many of us must face: universities, industries, and governmental relations. For that reason, I began searching and came across THM–primarily housed in innovation, which interrogates universities, industries, and governmental relations (see Figure 1) as a triple helix of evolving networks of communication (4).

THM has been used in innovation studies to explain how advancement comes as the result of a shared influence between the three helices. THM has been historically used to “explain the dynamic interactions between academia, industry, and government that foster entrepreneurship, innovation, and economic growth in a knowledge-based economy” (5). This model has rarely been used to interrogate sociopolitical aspects of the humans of which they largely impact. Galvao, Mascarenhas, Marques, Ferreira, and Ratten (6) conducted a systematic literature review of THM and found that it has been primarily used in four research clusters: (1) innovation and knowledge policies; (2) entrepreneurial universities; (3) business innovation strategy; and (4) triple helix stakeholders in innovation, knowledge, and regional development (p. 817). Wikberg-Nisson, Källhammer, Fältholm, and Abrahamsson (7) have utilized the model to understand how the three helices have been said to be gender neutral, contrasting this idea and emphasizing the need for an understanding of how the helices impact the way gender is operationalized. Delgado and Stefancic (8, 90) have also used a somewhat-similar framework (although they called it the Triple Helix Dilemma) to interrogate the ways that legal systems lead to a monolithic storytelling outcome for marginalized communities. Others (5, 9) have also tried to apply THM to empirical studies and educational spaces for students, though they tend to lean toward the Quadruple Helix Model (including civil society as a helix in the analysis) to focus on the freeing of minds for students. Outside of those iterations, there exists a gap in the application of THM regarding race, race relations, and critical consciousness pertaining to the way that race influences the experiences of people (6).

The current analysis autoethnographically adopts THM to explain how the helices (together and as separate entities) have collectively operated in a space where claims of race neutrality have been expressed–but not realized (10). Per contra, the helices have all aided in the creation, development, and reinforcement of monolithic ideas related to Blackness. Various books, articles, frameworks, and theories have been written (and shared) to explain the ways that the helices have historically impacted Black people (11–13), and through a THM analysis (14), I aim to conjoin those teachings to create a shared understanding. THM can allow for Black people to (re)claim Blackness by explaining the compound effects of racism from various locations. This is not to say that THM (as a model) is a direct mechanism of decolonization, but rather to say that it will be used as an analytical framework to make sense of the imbrication of conditions that produced my internalized racial anxiety when traveling to different countries. Travel and reflection on lived experiences sparked the identification of my racial anxiety, and THM was used as a framework to make that identification more palpable.

## Methods and materials: autoethnography

The usage of autoethnography as a method is central to the density, complexity, and cultural realities of Blackness–both in America and around the world. Autoethnography (as a method) “foregrounds the researcher's personal experience (auto) as it is embedded within, and informed by, cultural identities and con/texts (ethno) and as it is expressed through writing, performance, or other creative means (graphy)” (15). Autoethnographies are valuable in popular culture research (among other areas) and serve as a manner for researchers to provide a collective account of the ways that various phenomena, experiences, observations, texts, lifestyles, and participations impact understandings of cultural relations. This sense-making exercise allows for the lessons of lived experiences to inform the development, analysis, interpretation, and/or interrogation of cultural phenomena–while also allowing the researcher to offer guidance and wisdom to others in the process (15). In this way, I use personal experiences–coupled with cultural texts and literature–to make sense of my time traveling to a new country.

In this case, autoethnography is used to provide depth to my overall analysis–both of self and the world around me–by considering the larger impact of external aspects of my lived experience. To better make sense of my experience, I use cultural artifacts (i.e., music lyrics from Compton rapper Kendrick Lamar as epigraphs and analytic dialogue) alongside the work of James Baldwin (i.e., Global Blackness and finding freedom) as cultural nodes to make sense of identity, culture, and freedom for a Black sport professor exploring Blackness in global travel. I will blend the cultural works of Lamar and Baldwin with personal experiences, notes, and thoughts related to Blackness–all to more amicably explain the lived experience of decolonizing the mind and living “more free” as a Black man. Previous authors have used autoethnography (as a method) to explore their connections to racial representation (16), music and cultural texts (17, 18), literature (19), masculinity (20), and even Westernized associations (21). I aim to build on their collective contributions, blending all their angles into one beautifully Black tapestry of experiential understandings.

Due to the subjective nature of every Black experience, this work is not particularly concerned with notions of objectivity and researcher neutrality–as it is through my subjective experience and subsequent understandings that I attempt to provide a refreshing avenue of expression for others. A culturally-focused autoethnography of such can be used to explore how the development of Blackness, identity, belonging, and decolonization contains a multitude of complexities that can be better understood by also paying close attention to external forces that impact their construction. I hope to help other Black academics (or other Black folks in general) understand the ways that our various cultural aspects work in tandem to aid in the understanding of our experiences, while also plainly acknowledging that “it's not all in our head”. That is, there are very “real” aspects of our experiences (and things that happen to us) that shape/influence our construction of Blackness. Through a collective interrogation of the various aspects, we may begin to actively decolonize our minds by prioritizing lived experiences, agency, freedom, choice, and responsibility—in the immediacy of everyday life experiences (22). This reframing of mind and experience can aid in the creation of a bespoke understanding of our Blackness that is not tied to colonial notions of our existence.

It must also be noted that reflexivity (in addition to autoethnography) is central to my ability to make sense of experiences in Italy, especially as a methodological practice (as opposed to a mere reflective disposition). Reflexivity (as a methodological practice) can be understood as an ongoing process (23), explaining how my positionality as a Black-ademic sport professor (in the United States) influences my perceptions, interpretations of various encounters, co-production of meanings in settings, and ultimately frames my understandings of situational/social outcomes. Rather than bracketing the self, reflexivity (in this sense) is used as a manner of unearthing the multiplicity of limits, contradictions, revelations, and transformations that can emerge when Blackness moves across national/cultural boundaries.

## November 20, 2024—pre-arrival

In America, the color of my skin had stood between myself and me; in Europe, that barrier was down. Nothing is more desirable than to be released from an affliction, but nothing is more frightening than to be divested of a church. It turned out that the question of who I was was not solved because I had removed myself from the social forces which menaced me–anyway, these forces had become interior, and I had dragged them across the ocean with me. The question of who I was had at last become a personal question, and the answer was to be found in me. (Baldwin, 1992, p. xi-xii) (1)

My travels to Italy began first with excitement to be visiting a new land for personal exploration. Through my personal resonation with former basketball legend Kobe Bryant (24), I was overjoyed to be sitting on a plane headed to the country that Kobe spent his formative years in–especially considering how highly he spoke of his experience in living there for seven years (25). Admittedly, I wanted to believe when Kobe said, “people treat each other as equals there” (26), (para. 11), but as a Black man/professor, I could not dismiss what I also knew (academically) about this place. I could not dismiss how they have historically treated Black people in sport spaces, or even how Kobe also mentioned racism regarding his experience in growing up in Italy (27). I did not know what to expect, but I did know that life experience (coupled with my studies as a Black sport professor) had taught me to be on high alert in Italy. I thought of the intertwined nature of the Italian football world and that of politics, wherein their football clubs and supporters are intimately connected to political figures (28). I also thought of racial struggles faced by players like Mario Balotelli (a son of Ghanaian immigrants who is Italian) in Serie A (29), Samuel Umtimi vs. S.S. Lazio (30), Romelo Lukaku vs. Juventus (31), or general understandings of racism and player abuse in Italian football (32). I was excited for the new journey, but terrified at the idea of familiarity by way of racism. It would not be until much later that I would come to understand that the source of my apprehension was rooted in internalized racism and trauma–lived and inherited.

As the pilots announced to prepare for landing, I began to feel incredibly self-conscious about everything: my “self,” my body, my skin, my image…*everything.* I was confused, and admittedly conflicted, because I understand that I haven't been a competitive athlete since the day I “medically retired”. The fragmented athlete in me said to “prepare for war,” and the academic in me had the misfortune of rational agreement–because that is mostly what we (i.e., Black academics, henceforth known as Black-ademics) have been taught is the proper course of action in any uncertain position (13). America, Italy…anywhere, really. We don't belong, and people are intimidated by our Black skin in spaces (33), so try to acclimate and prepare to (if necessary) fight for existence. Some have said that the racism of Italians (i.e., the Ultras, supporters, or general public) is more attached to club rivalries (32), city rivalries (28), or sociopolitical differences as a result of changing political regimes within nations (34); I was simply hoping that those researchers were right. At the very least, I was hoping that my noise-cancelling headphones (and language barrier) would shield me from some of their comments. Music and sport were all I knew, and it was the only line of defense that I had. As we prepared for landing, I was hoping that this would be enough.

## (Re)imagining blackness—consciousness, representation, and misandry

In the early twentieth century, W.E.B. Du Bois' (35) popularized the theory of double-consciousness, wherein Black men are understood to encounter a two-ness of self when navigating everyday life in America. Furthermore, this two-ness of Black existence has also been called “the looking glass self” (36), considering the ways that Black people find themselves in a constant social-anxiety-like state while simply trying to live in peace without constant resistance from the world at-large. In the case of this article, Black men are said to exist in a constant two-ness where the man understands both how the America he lives in sees him and the ways that he understands himself within that America. Despite being coined in the early twentieth century, the state of double-consciousness for Black men is still prevalent and has been utilized in research related to academics (37), athletes (36), fatherhood (38), and even those who are differently abled (39). The wide-reaching nature of application for double-consciousness as a scope is quite fitting for many studies when considering the multifaceted nature of Blackness in the United States, but double-consciousness when exiting America and embarking on travels along the Black Atlantic (40) can make the process more convoluted. It is for this reason that I believe a more nuanced discussion is necessary.

In addition to double-consciousness, this analysis must also include conceptual understandings of people's reticence for Black males. The compound experiential effect of Blackness and maleness is one of unimaginable “weight,” as if Black men are holding the world on our shoulders. Furthermore, it feels as if Black men are constantly criticized/interrogated for dually being Black (relating to the color of our skin, subsequent heritage, etc.) and being men (regarding our biological sex, body type/size, subsequent roles, etc.). When considered together, these factors create an intersectionality-esque (41) understanding of the Black male experience. Unfortunately, this also comes with the negativity of “gendered racism” wherein the gender-specific ways that racism affects Black men's lived experiences create a compound effect of pain and strife that hinders overall progress (42). For this reason, the concept of Black misandry arose to explain the ideology and practices that marginalize, mischaracterize, oppress, and harm Black men (43). In particular, “Black misandry refers to an exaggerated pathological aversion toward Black men created and reinforced in societal, institutional, and individual ideologies, practices, and behaviors” (44). Black men also tend to face compound challenges, such as hyper-surveillance, social death (45), isolation, perceived criminality (46), association as “thugs” (47), stereotyped athleticism, and even anti-intellectualism. Furthermore, the ways that Black men and boys have been criminalized is “centered largely on monitoring and controlling their bodies across social institutions (such as schools and the criminal justice system), public spaces (stores, neighborhoods, and public roads and highways), and in the media as well” (46).

Black Misandry can not only affect Black men in the short term but can also have long-term effects on their socioemotional health, mental wellness, identity development, abilities to trust others, and overall well-being. In fact, Black men (myself included) must live with the unfortunate understanding that no matter what we do or where we go, the microaggressions and dislike will follow (91). As a result of such continuous pressure, Black men must negotiate their expressions of Black masculinity, responses to Black misandry, constant racial profiling, and hyper-surveillance by “performing” roles as a defense mechanism. Sometimes the performance is conscious, but many times the performance is more of a “codeswitch” where the Black man is simply adapting to the situation at hand to protect himself (or those he loves). “In effect, the Black male body is always already performing danger against whiteness, which informs white racial logics and a racist episteme that no black person can seek recourse to the visible as the sure ground of evidence” (46). It is through the scope of Black misandry that such responsibilities of the Black man, albeit unwanted, are at the core of all sociocultural interactions he must face–due to the negative ways that he is viewed by society.

As a Black sport professor, it became clear to me (along my travels) that the way I saw and understood my Blackness was just a manner of internal consternation–but was also affected by external forces as well. Understanding myself via the lens of double-consciousness featured a constant understanding of the ways that my Black maleness impacts people's perceptions of me, along with my perception of their perception. I admit that I experience this two-ness as a general train of thought throughout most days of life, but the realities of that experience have always become far heavier when I set onto planes and prepare to travel to countries outside of America. Unfortunately, the forthcoming trip to Italy would be no different. In fact, it would be the very trip that broke the lines of Blackness and forced me to consider the deafening fact that perhaps I had adopted some of the very aspects of racism that I had faced. Put differently, I realized that part of the racism I experienced had seeped into my personal walls and found their place as engrained aspects of my cultural realities.

This is not to say that travel (to or from anywhere) is a neutral site of observation that exists outside of the co-production of experiences, nor to say that Italy is a place to be monolithically known in totality. Instead, the reflections along my travels are utilized to foreground the limits of treating embodied movement as a study by acknowledging that I, too—as a racialized being whose presence (and body) also impacts the spaces in which it occupies/inhibits—actively play a role in shaping each interaction that I believe have reshaped me. Put differently, the trip to Italy allowed for a space for me to equally interrogate the ways that people *treated* me as a racialized other, the ways that I viewed those instances in real time, and the ways that my presence also impacted the experience for the other social co-creators in the space.

## November 20, 2024: arrival in Milano (not like us—Kendrick Lamar, 2024)

I stepped off the plane in hyper-Black-masculine fashion, resembling that of a footballer deplaning to prepare for a major match against an archrival. Admittedly, I was “performing” because I wanted to create as much distance (and admiration) as possible. Thompson (48) describes this as an “aesthetic of cool,” which is a culturally specific (of African lineage) mode of cultural composure, control, and endurance when under social pressure (p. 41). In my efforts to “keep it cool,” I paid close attention to my posture, movement, gait, exposition, and restraint as to give off the presentation of icy determination, self-control, and social equilibrium (48). I was the “cool cat,” and I wanted people to know that I was comfortable *visiting*—but I didn't quite want them to think I was a typical American. So, I West-Coast stepped off the plane, rockin a Black man outfit (i.e., a Black peacoat, Black pants, Black sunglasses, Black du-rag with matching skullcap, Black headphones, and 1984 Air Jordan's to complete the look). I made sure to sport my genuine brown leather carry-on duffle, as well as my Louis Vuitton backpack to ensure that the people knew I was a Black man of class and distinction (whatever that means). This was partially a performance, and partially a survival technique for self-regulation that has been shaped by centuries of Black aesthetic traditions of presentation, composure, and ancestral serenity (48). From the moment I breezed through customs and followed the *uscita* (“exit” in Italian) signs, I could feel the eyes of dozens of people watching me two-step my way through *l'aeroporto*. I knew of my racialized body, I knew how it might be perceived, and I understood that that was equally impacting the space for others around me—so I focused on what I could control and kept it cool. Icy cool.

I turned up my headphones and started the show.

My internal dialogue and walk with Kendrick went a little something like this:

*Kendrick: “Ay, Mustard on the beat, ho,*

Deebo any rap ni***, he a free throw”

(Lamar, 2024a) (49)

Check out these people on the left. They probably think I'm a footballer.

Oh, eyes on the right. That woman was definitely checkin yuh boy out!

*Kendrick: "Man down, call an amberlamps, tell him, 'Breathe, bro'”*

Nail a ni*** to the cross, he walk around like Teezo

(Lamar, 2024a) (49)

Flash a smile at this group of ladies. Yeah boyyy.

Throw the Black fist to the group of Black travellers waiting on their bags.

Just keep two-steppin, boy.

 We good.

  We almost outta here.

    *stops walking to hit a dramatic pause for this part of the song*

Kendrick: Beat your a** and hide the Bible if God watchin'

Sometimes you gotta pop out and show ni****

(Lamar, 2024a) (49)

Keep steppin,

 …pop out and show em who you are!

  and really bend this corner like Kendrick would…

 Scrrrrr. Hold up.

*two security guards approaching*

Stop dancing so much. Walk “right” and get your passport out, fool.

  Not too fast. Not too slow.

   (Just like with American police)

Kendrick: Wop, wop, wop, wop, wop, Dot, fu** ‘em up

(Lamar, 2024a) (49)

  Matter of fact, just stop before the fu** you up.

  In fact, don't f*** THIS up.

  You know nothing of international detainment.

  *Chill.*

  Maybe…it's time to act American?

  You know that blue passport is like gold…

*Kendrick: Wop, wop, wop, wop, wop, I'ma do my stuff*

(Lamar, 2024a) (49)

Nah,

  Maybe I can't “do my stuff” here.

  Pause the music, you actin' too Black,

   knowing damn well this ain't your space.

Step to the side with your phone in hand and act like you're searching for something.

 Anything, really.

  …just be cool.

     No matter what, it can't be much worse than America.

       …or, can it be?

***

## Of America, but not American…

The story of what can happen to an American Negro writer in Europe simply illustrates, in some relief, what can happen to any American writer there. It is not meant, of course, to imply that it happens to them all, for Europe can be very crippling, too; and, anyway, a writer, when he has made his first breakthrough, has simply won a crucial skirmish in a dangerous, unending and unpredictable battle. Still, the breakthrough is important, and the point is that an American writer, in order to achieve it, very often has to leave this country. (Baldwin, 1992, p. 5–6) (1)

It is one thing to be called American, but some people (like me) have sometimes taken offense to such a label because we cannot (in good conscience) accept a label of being a part of a country who enslaved, raped, pillaged, demoralized, defaced, demeaned, and outright dehumanized the ancestors of which we share a direct connection to. That is not to say I hate America, the place. After all, it is unequivocally my origin of life and, though I know that my lineage is rooted in Nigeria and Portugal, America is what I know; it is truly where I am “from”. Well, sort-of. I am dually a product of Mother Africa and the Europeans who raped her (50, 51), but unfortunately, I have no choice but to continue life as her informed child. I must live with her pain–as well as my own–and do my best to make her proud while knowing that our shared history is one ripe with pain, anguish, and a pleading for mere humanity. It truly hurts to harbor such painful remembrance, though as James Baldwin once wrote, “to be a Negro in this country and to be relatively conscious, is to be in a rage almost all the time. So that the first problem is how to control that rage so that it won't destroy you” (52).

Congruently (though not as severe), it is another thing to be deemed “Black” by some and “African-American” by others, because even though one can choose (personally, I choose Black)–the coding process by which one accepts the label is still based on the color of their skin as opposed to their/our character and abilities. Some black men may be unequivocally “Black” men (in most countries) but it's strange to recognize that the primary time some are considered “American” is when we are either serving as a signifier for prominent stakeholders…or no longer physically located within American borders. In fact, life outside of America is the primary space where many Black-ademic men receive categorization of American (by vocation, physicality, or even the navy-blue passport). What remains strange is, the treatment is often different than one receives in America. Obviously, one cannot expect to receive the same treatment from country to country, as that would largely ignore the specificity of varying cultures around the globe—and how they were shaped by specific forms of colonization and imperial conquest. Per contra, I mean to express that America's hatred for Black skin has led me to a depressive state of racial understanding where I now feel *on edge* anywhere I go. Put differently, it has become terribly difficult to remove–from my mind–the assumption that because America hates my Black skin, everyone else must hate it too. I have vivid memories of being treated terribly by people in America for being “Black” (24), but I also have other cases where I have experienced immense love and appreciation for being a “Black American” (53). It wasn't until I began to travel outside of America that I began to understand the complexities related to how the cultural demarcation of Americanness has led me to think differently about my own skin; I had no choice but to leave America, in search of deeper answers.

Much like James Baldwin, I simply “wanted to find out in what way the specialness of my experience could be made to connect me with other people instead of dividing me from them” (13). I didn't want the crimes of America to be the benchmark for which I would base my treatment of global people upon; I didn't want to do to others what America had done to me and judge the book by the cultural cover. But…how? For this reason, an expansion of the double-consciousness concept is necessary to understand the life of (American) Black-ademics who travel the world because no matter where we go, we are continually reminded of both our struggles along the Black Atlantic (40) and the ways that sport mirroring society has led to a constraining of our realities outside of American sport (and race) constructs.

To be a Black man is to continually know the struggle of mere existence (and societal value) outside of physical abilities (or, labor).

To be a Black male academic is to continually know that our intelligence will constantly be challenged, undermined, and will need constant reinforcement due to various stereotypes related to things like the “brawn vs. brain” framework (Brown and Williams, 2018) or “affirmative action hiring” (Hellman, Block, and Lucas, 1992).

To be a Black male academic in sport is to be constantly reminded that some people believe our value is situated in simply being “the Black professor” masquerading as a “dumb jock” who has learned a few big words but still cannot separate oneself from the physical-based sports that we compete(d) in.

We understand that sport (in this case) mirrors society, but is that the whole story? It has historically been a major challenge for Black men (particularly those of us who were/are athletes) to accept the label of being American because we have competed in a country where our Black bodies are largely valued for our physical contributions–while dismissing our mental abilities. Research related to Black athlete activism (54), ties to plantation logics (55), classism (56), racial capitalism (57), or even sexist dismissals regarding Black women (58) has been documented, focusing on the ways that America has mistreated Black people as a whole. Much of this work has been achieved using scopes like Critical Race Theory (59) and Black Masculinity (60), but the byproduct inadvertently created a dangerous mental attitude where some Black men then come to believe that the world is against them.

As a Black man, you're damned if you do…and damned if you don't.

…but…damn.

Perhaps there are more aspects of this analysis that deserve inclusion, and because I don't believe that it's as simple as reverting to aspects of double-consciousness along the Black Atlantic, I instead decided to look outward. That is, America has imprinted on me a very confined way of thinking about global cultures and the ways they might encounter my presence (and Black body)–but it does not do so using the same avenues of oppression. Instead, it may be more amicable to consider the various sectors of America that have influenced my current understanding of culture, society, Blackness, and self. In this case, I feel that I have been most influenced by government specifics, industry aspects, and university understandings–though I'm not sure which one has had a larger impact. Fortunately, the Triple Helix model of analysis affords a framework to interrogate the compound impact of all three on outcomes and experiences. Though this model has not been used directly in reference to racial experiences and representations, I believe that the adoption of this tri-fold model of influence aids to best explain the ways that the construction of Blackness is impacted, molded, and reinforced by all three simultaneously (11). In what follows, I attempt to explain the ways that this collective understanding not only aids in my interrogation of global Blackness but also serves as a way for me to decolonize my mind and finally experience a freeing of racial confines while abroad.

## November 28, 2024: poncino (alright—Kendrick Lamar, 2015)

I stood in Piazza Garibaldi with a friend after some shopping in Corso Italia in Pisa, Italy. We fancied a drink, so we decided to visit a local place and have poncino (a small, Tuscan drink served warm with coffee and liquor). Once we ordered the first round, I noticed that the fellas behind the counter were paying close attention to me. At every turn, they were slightly eavesdropping on our conversations and paying attention to my reactions regarding their music choices, as if they were somewhat seeking my reaction (or approval). My consciousness informed this awareness, which was confirmed by my friend (who is local and was also paying intimate attention to the setting) laughed at how seemingly oblivious I was to their attention. Little did she know, I wasn't oblivious; I was performing.

*You really do have a crazy impact on people here. Did you know this?*

What do you mean? I feel like people are always looking at me strangely. What impact though?

(thought to self: it's probably because I'm Black and Italians don't like Black people of my stature)

*Well, I notice that they have been staring at you since we arrived.*

* Do you know why? I think I know…*

*I sharply responded*

Easy. It's because I'm Black and I look this way (gesturing to my face and body)

 That has been my primary concern since booking this trip

  …the racist people here

   you know, like the Ultras…

    Everybody knows they don't like Black people.

I then proceeded to tell her about my knowledge related to the Italian Ultras (34), crimes against Black footballers in Serie A (32), and even the Italian inclusion with the transatlantic slave trade centuries ago (61). It is hard to dismiss the challenging ways that the Ultras have been explained within sport contexts, especially when recognizing the influence of global media (62).

She seemed confused:

*Oh.*

* …okay yeah, I'm…*

*I'm sorry. I didn't know this…but is that what you feel here?*

* Do you think all of Italy is just a bunch of*

*  …how you say…*

*    “Ultras?”*

  …*because*

*That's not what I was saying at all.*

* In fact, I was saying that they love you here.*

*  I mean, just listen to the change of music…*

(at the time, they switched the music from Italian pop to American Rap; Kendrick Lamar's “Alright” was chosen and the volume increased)

*You can even turn around. They’re staring at you right now,*

* Smiling. Dancing. Following your energy. They love you.*

*  It seems like they're trying to be friends with you.*

Just then, I turned around and noticed that she was right. The guys behind the counter were all looking at me with joy, admiration, and celebration as I rapped the words to the song.

Kendrick: Alls my life, I has to fight, n****

*Alls my life, I—*

*Hard times like, “Yah”*

*Bad trips like, “Yah”*

*Nazareth*

(Lamar, 2015) (63)

***

I then had a moment of reflection (and confusion). Perhaps I had it all wrong. Perhaps I was treating Italian people writ-large with a stereotype I learned through studying sport and understanding the hatred that Black footballers had experienced regarding the Ultras. Perhaps, I was unfairly judging the many for the actions and words of the few–which is exactly the stereotyping that I wished other people would stop doing regarding people who look like me. I proceeded to explain to my friend that I grew up in a place that can be quite hateful and unforgiving, along with living (and working) in a field of study that can share those same characteristics (because sport is said to exist as a microcosm of the social world). Based on my academic teachings/understandings regarding American sport (and life), I needed her to understand that my “stereotyping” was based on a vast record of historic consternation. No matter how short-sighted it had seemed, I tried to explain that it was more of a manner of survival than hatred or stereotyping.

***

Kendrick: I'm f***** up, homie, you f***** up

(Lamar, 2015) (63)

I sipped my poncino (which the employees gave us “on the house” after a kind embrace about their music) and said to her quite directly:

In American sports, if you are wrong about your opponent…you lose.

In American life (for people like me), if you are wrong about your judgments of people, you die.

It's simple: Black people only get one shot at this. We don't usually have the luxury of giving the benefit of the doubt.

People ask why we are always defensive, and as a Black man in sport, I say two things:

1) defense wins championships

2) defense keeps you alive.

I just want to stay alive and protect those I love so they can do the same.

It's not personal, we just don't have time for smiles when we have experienced so much pain.

She sat for a moment, puzzled, then said,

*I understand…and I can never know what that must be like…but can I ask a different question?*

Sure (noticeably agitated; sharp response).

*When exactly do you let yourself feel alive…if you are always guarding yourself to ensure you don't die? I mean, is there ever a point that you just live for the sake of living?*

*I don't mean to be obtuse because I totally understand your point.*

 *…well, I don't, because I can't, but*

  *I'm just trying to understand*

   …*aren't you tired? That sounds exhausting to have to live like that*.

*Kendrick: But if God got us, then we gon' be alright*

(Lamar, 2015) (63)

I was silent, but thought to myself:

 I hope someone “got” us.

  I mean, “God” didn't help my ancestors on the transatlantic voyage

  So why would that “God” help me? Protect me? Love me?

 What the fu** ever.

   Yes, I'm exhausted,

    …but at this point, I don't know how else to be.

  I ain't got time to believe in being saved, so it's whatever.

   God or not,

    …we gon be aight.

***

She was right, I just didn't want to admit it. Many of us (i.e., Black men) are dying to live freely without having to worry about racial stereotypes, misandry, or general mistreatment; we are pleading for release from the confines of racial battle fatigue (64). We have been conditioned to understand that life will always be a battle along racial lines; the struggle that must be (65) and subsequently become hardened in the belief that no other reality can exist. At times, it feels as if we are simply living to die because we are constantly made to choose “the lesser of two evils” by allowing the constraints placed upon us to dictate our expressions of self.

This is sometimes exacerbated in the sport space, primarily because we have the double-consciousness of being a Black man in America *and* the equal consciousness of being a Black man in sport. The challenge then becomes: how do we shed this? How do we allow ourselves to live while also knowing what we know? How do we allow ourselves to freely exist when we know that our ancestors gave people the benefit of the doubt and were taken advantage of in the same way (66). Personally, how do I also allow myself to “just be” when that level of comfortability in America can get you killed, drugged, or publicly crucified. These are all choices that some Black men make, consciously or subconsciously, every single day. Our double-consciousness, understanding of treatment related to Black masculinity and Black misandry, and knowledge of how Black men have been treated in sport spaces makes this a conundrum that continues to plague many–sometimes for a lifetime (67). I refuse to live like this for the rest of my life, so it now comes time to determine just how I got to this point in the first place.

## Triple helix model and the black struggle

My experiences as a Black male sport professor in Italy was truly liberating, as it opened my eyes to the compound effects of my Westernized upbringing (13). Never had I considered that not only was everyone not out to get me (because I'm Black), but also that the very reason I thought this way…was a compound effect. I studied at university for ten years, participated in various industry activities, and studied law and its relation to governmental relations (both in America and abroad). I read various cultural texts (50, 68, 69) that have spoken intimately about the damaging effects of America's colonizing crimes against Black bodies–but never had I paid attention to the ways that most authors have anthropomorphized America some sort of monolithic being. America, in all his crimes and shortcomings, is better understood as a brotherhood of siblings that equally influence the realities of anyone outside of their white capitalist patriarchal structure (12). It is not my intention to hold anyone's feet to a fire or point fingers to say that America is unanimously a bad place. Instead, I am simply calling a spade a spade (70) and acknowledging the ways that the helices together have created a tumultuous environment for those of us who don't fit (as free, valued, and contributing actors) into their power structures. In what follows, I had no choice but to trace the roots beneath the feeling—and examine the structures (universities, industries, and government systems) that have aided in their production.

### Universities

I will begin with universities, as that is the primary location where myself (and many others) are trained, educated, socialized, and ultimately evaluated. We go to university to get an education and learn more about the world that we had been previously unaware of. The density of my experience with universities is exacerbated by the fact that it is also where I am employed and continually evaluated as a tenure-track professor. I take great pride in my vocation, but it is certainly not without its struggles when I am continually reminded that I am a *Black* professor–not just a professor. It is also not lost on me that universities have not always been places of privilege for Black people. Not only were Black people not allowed to learn (and were sometimes killed for learning) simple skills like reading and writing, but we were systematically (and legally) not allowed to attend major universities before our legal emancipation in 1865 (71). It would take the emergence of Black colleges and universities, largely because of the Black Codes in 1865–1866, to truly begin our advancement by way of higher education (59). Even then, universities were largely hesitant to allow Black people to study alongside their white student bodies and major changes would not come until notable civil rights legislation came to fruition (discussed below). Due to the constrained history of universities and their treatment of Black people, it comes as no surprise that problems related to belonging/non-belonging, surveillance, advancement, and overt gatekeeping have negatively impacted Black people–in and out of education.

This is a problem that is one of the easiest for me to notice and take heed to, specifically as an academic professor at a historically white institution (HWI). Not only has the Black struggle for education been well documented in general, but it has been explored from various angles related to the experiences of students (72), athletes (73), administrators (74), and professors alike (75). Universities exist as institutions of higher education and learning, but problems arise when the stories and histories of Black people are written and taught to Black people–from the angle of the oppressors (12). Furthermore, I still see these problems daily. I see the ways that universities are now barred from teaching concepts related to diversity, equity, and inclusion (DEI) due to ongoing legislation in the United States. I also see the ways that racism and whiteness inform these decisions, where white leaders of majoritarian structures continue to remain (willfully) blind and ignorant to the ways that their politics have oppressed Black people (92). A collective history could easily be written regarding American universities and their crimes against Black people, but the overall point is to note that for many Black people, universities have been one of the most complicated aspects of our development due to the fact that many of us are still made to feel like intruders in spaces that are said to be places for the development of cultural consciousness. Unfortunately, universities have been a continual site of reinforced racism and other forms of oppression (i.e., classism, racism, heterosexism, etc.), and until the powers that be decided to acknowledge and repair this structural consistency of marginalization–the Black mind will continue to experience negative impacts.

### Governmental relations

Globalized racial governmental development and subsequent policies specific to the American government (over the years) cannot be ignored as a major force of oppression for Black people in the country (and abroad). Unfortunately, the effects of America's governmental efforts have been seen to impact many Black people (and plenty of other groups) both domestically and internationally. Regarding Black people, this racialized regime precedes the official development of the land as a colonized country itself, as race became a social construct alongside the establishment of enslavement efforts–as early as the fourteenth century. Early Americans (as transplants from Europe) adopted racialized policies and operational strategies from the beginning, using race (and religion) to justify their crimes against Black humanity (76). Aside from the very notable crimes related to the transatlantic slave trade and subsequent mistreatment of enslaved Africans whose physical labor (and reproductive labor) would become the primary avenue for the building of America itself, it must be noted that America did not work alone. Numerous European nations (inclusive of Italy) were also responsible for the 400 years of tyranny experienced by enslaved Africans along the transatlantic slave trade (68). Furthermore, the “founding fathers” of the country also made sure to include aspects of racial marginalization into the founding documents themselves. Consider for example, Section 2 of Article I of the United States Constitution, which established a three-fifths compromise wherein enslaved Africans were not seen as a “whole person” (or remotely human) and instead counted as three-fifths of a person primarily for taxation purposes (36). From there, the American government would continue its mistreatment of Black people through its legal system, and it wouldn't be until the famed *Brown v Board of Education* decision in 1954 that the public education system would (slowly) become desegregated. Although this wouldn't completely free Black people and allow them to attend white schools (largely because of states' rights), it would be a landmark decision that would aid in the advancement of Black people regarding education.

Much of the Black struggle in America has come because of the government and its domestic (and international) relations. At various times in America's history, it has been illegal for Black people to read, write, own houses (or anything really), vote, marry, date outside of race, and even use the same public facilities as white people. There have been copious articles, books, theories, and frameworks (historically and contemporarily) that have been developed to explain the impact of the American legal system on the Black experience, though none are seemingly more explanatory than that of Critical Race Theory (CRT); (77). CRT, which doubles as a theoretical framework and a movement, began as a manner of study when early scholars such as Derrick Bell Jr. noticed a lapse in the ability of Critical Legal Studies to acknowledge the racial components laden in the legal system (78). This would be one of many analyses that would grow to call attention to the racialized legal system in America, so much so that the government (and plenty of member states) essentially declared war on CRT; at least 28 states have banned (or restricted) the teaching of CRT in classrooms (79). The American government's impact has not stopped there, as contemporary efforts have grouped DEI into its conceptual attack and either banned or restricted the teaching (and operation) of DEI (and subsequent policies) from being taught (and exercised) in schools. To date, at least 22 states have passed legislation restricting DEI-related aspects in schools–inclusive of the state in which I currently reside (80). Lastly, there has been a consistent elimination (or combining) of programs like Africana/Black studies, along with Women's, Gender, and Sexuality studies programs across the United States—which further marginalizes/erases the study of racialized experiences for Black people [among others; (81, 93)]. The constricting of teaching has not only had profound effects on the student/teaching experience, but it also serves to inform the ways that their funding bodies, policies, mandates, and rules have continually worked against the advancement of Black people and their experiences. The American government's historic marginalization of Black people (inclusive of our stories, perspectives, and experiences) is clearly evidenced by centuries of policies, laws, and rules (spoken and unspoken alike) that shape who gets access to resources–and who doesn't.

### Industries

It is no secret that America has placed his value on the power of the almighty dollar. In reference to the explanation of the rap group Wu-Tang Clan, “cash rules everything around me”. America's emphasis on capitalism is heavily known and documented across the globe, so much so that many global nations resist American inclusion in globalization circles out of fear that capitalism will tarnish their cultures (82). America's capitalistic framework was created alongside the inception of the nation, as this was the primary reason that America stole enslaved Africans and dehumanized them for the exploitation of their physical (and less discussed, mental and reproductive) labor (68). From there, America would develop industries as secular spaces where various iterations of labor have been valued, commodified, and even constrained–depending on their impact on revenue generation. Obviously, there are plenty of industries that are necessary for people to make a living, and within those industries, people are known to assume many roles; from academic professors and administrators to employers, companies, corporations, non-governmental organizations, and even smaller organizations. The problem then comes in when we think back to the social construction of race and the ways it has been used to control Black inclusion in industries. The first “vocation” for Black people in America was one that they did not choose, nor did they enjoy: enslavement. Once the Emancipation Proclamation was passed in 1865, Black people were “freed” from enslavement and henceforth allowed to participate in society as “free” people on the labor market (allegedly). Although this would serve as symbolic progress for Black people, many Black people would still not be able to work or attain jobs outside of sharecropping and other physical labors, as the hangover of the country's workforce politics would continue to impact Black advancement. Many of the newly-freed enslaved people struggled to find jobs, and with the development of Jim Crow laws, the overall experience would become far more difficult. It would not be until the landmark *Civil Rights Act of 1964* that industries were no longer allowed to deny employment based on race (36). Even though this legislation was paramount in increasing employment opportunities for Black people, the systemic racism embedded within industries has still made it difficult for contemporary Black people to find opportunities and move toward advancement.

The laden racism within industries is an undeniable aspect of industries that has had lasting impacts on the Black experience. Blackness in general is at times regulated, constrained, read, and (de)valued in many ways within industry settings. Many Black people have even been made to feel less than for lacking (so-called) professionalism in various industries, with conversations related to hair, dress, style, language, and overall fit (to name a few) have been used to deny Black advancement (or, mere existence) in various industries. The reliance on a politics of respectability directly targets Black people in and beyond industry spaces (83, 84), which manifests as another way that opportunities for advancement and growth (for Black people) have been constrained; I am no exception to that reality. For example, I was forced to cut my “locs” (a historic Black hairstyle featuring long, braided strands of natural hair) before graduating from university because the hairstyle was deemed unprofessional by many whom I had interviewed with. Contrary to popular belief, this manner of controlling Blackness (and culture) is something that many Black people still experience, and though it may be of lesser degree in the age of social media (purely due to exposure and fear of backlash), Black people must still be aware of the reality that they may have to change their hair or speak differently if they want to be included in particular industries. Hair, for Black people, has always been shaped by the contemporary politics of the time, and even with interventions such as the Creating a Respectful and Open World for Natural Hair (CROWN) Act, discrimination against race-based hairstyles, particularly those created, and worn by Black people (e.g., locs, twists, braids, afros), remains a central marker of our place in society (85). Fortunately, some industries have allowed for more cultural diversity, and this has even led to the development of employee resource group where Black people can create their own and find solace by connecting with others who look like them (and share cultural understandings), within (and outside of) their companies. American industries are vehemently focused on revenue generation, and because they have been known to choose money over cultural diversity (and outright humanism), industries remain a primary location where Blackness is negatively impacted.

## The breakout (All the stars—Kendrick Lamar ft. SZA, 2018)

The utilization of THM in this cultural autoethnography is paramount to understanding why I experienced such convoluted trepidation while traveling in Italy. My understanding of governmental relations, universities (and their teachings/impacts), and industries (as a sport professor) had collectively aided in my (admittedly troubling) recognition of my own internalized racism. In America, I have always been called a *Black* professor (not just a professor); a *Black* man (not just a man); a *Black* athlete (not just an athlete). Through a THM analysis, it might be more plausible to understand why everything I had experienced until this point had led me to think that everyone hated Black skin–and that I needed to be on guard no matter where I went. It would truly be a conversation I had with my friends in a car ride on the way back to our flat that I would see how my internalized racism (and subsequent understanding of my colonized mind) had impacted my trip from start to finish. I rode in the backseat of the car, headphones on full volume (listening to Kendrick, of course) and gazed out the window along the *Arno River* in Pisa when my friend (who was driving) began staring at me in the rearview mirror. I took out one earbud, though I could still hear Kendrick's poetry in the other.

*Kendrick: Tell me what you gon' do to me*

*Confrontation ain't nothin' new to me*

*You can bring a bullet, bring a sword, bring a morgue*

*But you can't bring the truth to me.*

(Lamar and SZA, 2018) (86)

(I stared into the sky, listening to “All the Stars,” preparing for what's to come).

He begins:

*So, my friend, tell me:*

* Now that you have been to Italy for quite some time,*

*  What do you think of the Italian culture?*

Aye man, it's been cool!

I've enjoyed myself overall!

La dolce vita, right? What's not to love?

Kendrick: Fu** you and all your expectations

*I don't even want your congratulations*

*I recognize your false confidence*

*And calculated promises*

*All in your conversation.*

(Lamar and SZA, 2018) (86)

(Admittedly, I waited for his response; on guard and waiting for the other shoe to drop).

*Okay, fair enough! I love that.*

* Next question:*

*  What are the stereotypes of Italian culture that you expected to see*

*   …and was it exactly what you expected?*

(Damn, here we go. I could lie, but do I need to?).

*Kendrick: I hate people that feel entitled*

*Look at me crazy cuz I ain't invite you*

*Oh you important?*

*You the moral to the story?*

*You endorsin?*

Mothaf***a, I don't even like you.

(Lamar and SZA, 2018) (86)

Well, I can't say it was what I expected.

On one hand,

I was told that Italians truly love their wine, pasta, and football clubs.

…and part of that is true;

I have never eaten so much pizza, pasta, or bread in my life!

*Kendrick: Corrupt a man's heart with a gift*

*That's how you find out who you dealin' with*

*A small percentage who I'm buildin’ with*

(Lamar and SZA, 2018) (86)

(We all share a warm laugh, but I can see his eyes in the mirror…waiting expectantly for me to continue. I felt comfortable, and he gave me no reason to hate him, so I continued).

 But…

  I expected to see rampant football fans.

  …and rarely saw any.

  I expected to deal with a lot of racism.

  …and never felt any.

  I expected people to heckle me and give me a tough time…

(I see his eyebrows raise as he looks at me in the rearview mirror)

*Wait, so you expected to not like this place?*

Unfortunately, yes.

 I was prepared for the worst…

(He cuts me off before I could say more).

*It's interesting that you prepared for the worst,*

* …because many here actually think the Ultras are misrepresentative.*

*  I mean,*

*   …some even believe they are the worst representation of Italian society.*

*Kendrick: I want the credit if I'm losin or I'm winnin*

On my mama, that's the realest sh**

(Lamar and SZA, 2018) (86)

***

“The worst representation of Italian society” was an unexpected way to hear of such a group that has historically been central to many sociopolitical (and sport) understandings since the late 1960s (34), but again–he was right. I had to take credit for the ways that I thought Italian people would be, purely based on my THM understanding. We continued our chat as my friend kindly returned us to our flat, but my mind was admittedly not focused on the conversation because I was more focused on the new information that had just been empirically gifted to me from a local. That conversation was followed by another conversation with my friend (with whom I shared poncino), who shared that they had talked to a colleague about my dilemma. When I told my friend about how my conceptual (informed by the collective THM) understanding of Italian society (along with confusion regarding my teachings in sport spaces) was now shifting greatly, she laughed.

*Well, yeah…I'm glad you're listening and experiencing this,*

* …because I was scared that you would let that sports stuff ruin your trip.*

*  Not all Italians are like that. Most of them I know want nothing to do with sports.*

(I responded, admittedly confused)

 Wait, really? So nobody likes sport that much? How?

  It is integral to many political movements and understandings of Italian culture.

*I don't doubt that, but I asked a colleague about it at coffee today and she said,*

*“Yes, I’m glad he gets to see the real Italy and Italian culture,*

* …I mean, why do you think they actually built the colosseum?*

  We all think its so the sports people have a place to go and k*** each other

*   …while leaving the rest of us in peace. They're just crazy.”*

***

Here, I do not intend to dismiss the power of sport within any particular society and culture, nor do I believe that this person's comments were anything other than satirical humor based on anecdotal experiences; it was an interesting counter-narrative to everything I had heard about this group. Once again, I faced a conundrum regarding what I thought I knew, vs. what I am now gathering empirically. Granted, the power of storytelling is central to critical research and has been a great avenue for expression regarding the development of intercultural understandings in spaces related to various cultural locales. With that understanding, it is not surprising that the narratives of “local” people are different from that of a majoritarian understanding of the role of sport in society writ large. What is surprising is the way that as a Black researcher, I found myself trying to dismantle the master's house using the master's tools (87). Put differently, I discovered that I too was holding the Italian society to the stereotype of *Ultras* without giving the people a chance to prove me wrong; I was theoretically blaming the many for the subsequent actions/understandings of the few. Once I came to this understanding, and its congruence with my life experiences, I began to cry.

How could I be judging Italian people before even giving them a chance (figuratively what America has done to me)?

Is it because of my double-consciousness?

Is it because of university understanding? Industry pressures? Governmental constrictions?

Herein, lies the triple helix dilemma…

## A first-class realization, en route to America—(Man on the garden—Kendrick Lamar, 2024)

Once I was able to accept my role–as distinguished, I must say, from my “place”–in the extraordinary drama which is America, I was released from the illusion that I hated America. (Baldwin, 1992, p. 5) (1)

I put my headphones on, suited up in my typical all-Black fit, and prepared to return to America. I boarded my flight early because I booked an upgrade to first class, and though it felt good to be the only Black person sitting in first class, I was still partially troubled. I had flown first class before but based on the realizations I had experienced while being in Italy, this time felt different. I felt…out of place, but not exactly *out of place*. I quickly made my way to my seat (where I was greeted with a beverage), sat down, and assumed my usual cross-legged position as we waited for others to board the plane. My people back in America had been continually messaging me to listen to a new album from Kendrick Lamar, so I cued up the album and began listening for a few seconds. As I watched the other passengers board and admittedly stare at me with confusion, I paused the music because I wanted to hear if anyone added commentary to complement their confused stares when seeing me amongst the elite of first class. Surprisingly, it was just like that day I was sippin poncino, and immediately I was at ease; relaxed. There were many looks, but no stares. Mostly, people smiling, nodding, and offering a quick “Ciao” before continuing their journey. I stared through polarized lenses as people minded their own business and made their way to their seats. At that moment, I realized that the early stages of my newly decolonized mind were bearing fruit; my internalized racism had been significantly reduced from the continued “fire next time” to a mere simmer. I was overjoyed, but still uncomfortable; the newness perplexed me.

Why am I assuming they would be surprised that I'm sitting here?

 (Internalized racism rearing its ugly head)

Why am I assuming they would be confused?

 (Internalized racism rearing its ugly head)

Why am I assuming…

 That they give a damn at all?

 (Internalized racism, leave me the fu** alone!)

(I shook my head, laughed, picked up my phone, and pressed play).

*Kendrick: Twice emotional stability*

*Of sound body and tranquility, I deserve it all*

*Like minds and less enemies*

*Stock investments, more entities, I deserve it all*

(Lamar, 2024b) (88)

(I nodded my head, the hip-hop way, as the song continued)

Man, he's right.

 I deserve it all.

  We all do, all of us.

   We deserve this.

  We deserve love, joy, and happiness.

   We deserve to live outside of the confines of colonized minds.

    We deserve…it all.

     I…deserve it all.

In a moment, everything flashed before my eyes and I thought of my mother; her struggles, trials, and tribulations. I thought of how much she must have sacrificed for her son, the athlete-turned-professor, to be leaving the country of Italy in a first-class seat, headed back to America. The lyrics of the song rang like church bells in my head and sparked a fury of emotions and goosebumps because, for the first time, I felt that I was in control of my experience…and that I shouldn't waste my mother's sacrifices by believing in the tropes of Blackness that had been placed upon me.

*Kendrick: Put a smile on my mama*

*Good health and good karma*

*Yeah, she deserves it all*

(Lamar, 2024b) (88)

(Pause the music. I need a minute with my tears).

***

This trip to Italy taught me a great deal, and as a Black sport professor, I learned the power of actively working to decolonize the mind of expectations related to Blackness, letting go, and letting love. That is not to say that this is a generalizable style (though parts of my reflective analysis can certainly be useful), nor to produce generalizable claims about Italy, Blackness, or Europe writ large. Instead, my reframing of mind was transformed through movement to-and-through spaces previously unbeknownst to me. Black-ademics can allow the movement and navigation of spaces to support expansive understandings of how we too transform the spaces we enter—just as we may feel they transform us. I (re)gained my Blackness in Tuscany, and I couldn't help but embrace America for its gifts and curses. I am only this me–Black or otherwise–because of everything I had experienced until this point. I could not dismiss the depth of my knowledge (and feeling) of despair for America, but at the same time, I realized that I could take back my power and use my experience as an avenue for Black (re)imagination (10). Although I will not dismiss the existence of racism experienced by sport (and non-sport) people who look like me, I have come to the understanding that travel, decolonization of the mind, and the release of internalized racism is of utmost importance to any group of people (in this case, Black folks) who wish to be embraced and allowed to freely exist with respect to their cultures, communities, and customs. Most of my conceptual understandings of people, cultures, and customs have come by way of experiences in America, but it would be foolish to allow such a monolithic scope to dictate how other cultures are defined–or even how to define them oneself. In fact, I imagine it is this precise reflexive entanglement that exposes the need for actively reframing our struggles and standpoints—moving from an eternal struggle with “colonized” frameworks, toward one of freedom and cultural liberation (22). Perhaps this means that those of us on the Black Atlantic (particularly the Black people in America) must leave the confines of America's shores to truly find self and create Blackness in life and meaning–or leave behind the notions of “creating Blackness” and “decolonizing” altogether; a complete reframing of minds conditioned by colonization, toward agency and responsibility to think differently (2). This is not to say we should harbor a hatred or disdain for America, but rather a reminder that our experiences as Black folks do not have to be defined by the chains of colonial structures. Blackness can mean whatever Black people want it to mean; Blackness is not a monolith. We must get away from mundane explanations and continual defense of self and instead step into the beautiful world of decolonized thinking where we don't have to allow projected titles, layers, and structures to define us.

Through a careful analysis of Blackness through the Triple Helix model, I have learned that the problem may be rooted in my own adoption of principles, titles, and expectations laid upon me. I acknowledge that much like many other Black people, breaking the chains of internalized racism (and subsequent colonized thinking) is no easy task. Through a THM analysis, I believe we can at least get to the point where we remember that we have the freedom to choose otherwise. It is not easy, and it will take a great deal of personal reflection to get to the promised land, but it is worth the journey. THM can be helpful to explain the variation of structures that shape our consciousness, but it must be through our own embodied experience (beyond the United States and its racial foundation) that we can reframe our understanding ways to exist beyond resentment for historic challenges and major stakeholders. We can't change how universities, industries, and governments operate, but much like our ancestors, we can make it do what it do (70). Put differently, just because we are told to believe certain ideas about Blackness and are subsequently influenced to operate a certain way–doesn't mean we have to. If we break these larger helices down, they are nothing more than figurative lenses of looking at things. Furthermore (and much like other glasses), perhaps it is time to take them off…and see the world as we wish. Granted, I am not sure that this is possible (regarding an overhaul of the deep embeddedness of whiteness, and more), nor do I know what it will mean if operationalized. This could mean that Black people may have to leave America (temporarily or in totality), or perhaps distance ourselves from the critical study of sport (temporarily or in totality). Maybe…both require our exit (temporarily or in totality) for us to truly explore our Blackness outside of the confines of the triple helix. That is not to say these dynamics don't exist in other places, or to say that all non-American spaces are somehow colorblind in sociological operation (13). Rather, this is a scope to suggest that Black people be able to start over and potentially create a new framework of consciousness that doesn't start with immediate racialized differentiation based on a life spent in the triple helix framework of damn near everyone–inside and outside of sport–mistreating/exploiting us for our Black skin/bodies.

This battle cannot happen alone though, as institutions (widely defined) could also be a source of support regarding such a major cultural shift. Institutions must be better in terms of preparing Black people, while also working to actively debunk stereotypes. Changes in industries and governmental actions are also needed, as Black people need more space (and support) for self-actualization beyond the stereotypical depictions of Blackness. This could be by way of pedagogical changes and teaching such as the community cultural wealth model (89). This could also happen by way of involvement in government spaces where change can come by way of the legal system (considering this has been the primary location of operationalized oppression). Together, each American helix has a role in supporting the development of Blackness for its citizens, but if not, we the Black people must stand together and choose (for ourselves) to identify and debunk the idea of monolithic Blackness. We must define it for ourselves, support each other in our work to dismantle the structures that keep us hidden, and if need be, find a place where we can go and be celebrated–instead of tolerated.

“We travel not to escape life, but for life not to escape us”—Anonymous

## Data Availability

The original contributions presented in the study are included in the article/Supplementary Material, further inquiries can be directed to the corresponding author.
